# Validation of a small-area model for estimation of smoking prevalence at a subnational level

**DOI:** 10.18332/tid/169683

**Published:** 2023-09-01

**Authors:** Carla Guerra-Tort, Esther López-Vizcaíno, María I. Santiago-Pérez, Julia Rey-Brandariz, Cristina Candal-Pedreira, Leonor Varela-Lema, Anna Schiaffino, Alberto Ruano-Ravina, Monica Perez- Rios

**Affiliations:** 1 Área de Medicina Preventiva e Saúde Pública, Universidade de Santiago de Compostela, Santiago de Compostela, Spain; 2 Servizo de Difusión e Información, Instituto Galego de Estatística, Xunta de Galicia, Santiago de Compostela, Spain; 3 Servizo de Epidemioloxía, Dirección Xeral de Saúde Pública, Xunta de Galicia, Santiago de Compostela, Spain; 4 Epidemiología y Salud Pública, Centro de Investigación Biomédica en Red (CIBERESP), Madrid, Spain; 5 Health Research Institute of Santiago de Compostela (IDIS), Santiago de Compostela, Spain; 6 Departament de Salut, Direcció General de Planificació en Salut, Generalitat de Catalunya, Barcelona, Spain

**Keywords:** small-area analysis, prevalence, smoking, health surveys

## Abstract

**INTRODUCTION:**

Small-area estimation methods are an alternative to direct survey-based estimates in cases where a survey’s sample size does not suffice to ensure representativeness. Nevertheless, the information yielded by small-area estimation methods must be validated. The objective of this study was thus to validate a small-area model.

**METHODS:**

The prevalence of smokers, ex-smokers, and never smokers by sex and age group (15–34, 35–54, 55–64, 65–74, ≥75 years) was calculated in two Spanish Autonomous Regions (ARs) by applying a weighted ratio estimator (direct estimator) to data from representative surveys. These estimates were compared against those obtained with a small-area model applied to another survey, specifically the Spanish National Health Survey, which did not guarantee representativeness for these two ARs by sex and age. To evaluate the concordance of the estimates, we calculated the intraclass correlation coefficient (ICC) and the 95% confidence intervals of the differences between estimates. To assess the precision of the estimates, the coefficients of variation were obtained.

**RESULTS:**

In all cases, the ICC was ≥0.87, indicating good concordance between the direct and small-area model estimates. Slightly more than eight in ten 95% confidence intervals for the differences between estimates included zero. In all cases, the coefficient of variation of the small-area model was <30%, indicating a good degree of precision in the estimates.

**CONCLUSIONS:**

The small-area model applied to national survey data yields valid estimates of smoking prevalence by sex and age group at the AR level. These models could thus be applied to a single year’s data from a national survey, which does not guarantee regional representativeness, to characterize various risk factors in a population at a subnational level.

## INTRODUCTION

The most recent Spanish National Health Survey (NHS) (*Encuesta Nacional de Salud/ENSE*)^1,2^ was carried out in 2017. Using the NHS, the prevalence of different health determinants can be estimated by sex and age at a national level and by sex at an Autonomous Region (AR) level. Based on the 2017 data, smoking prevalence in Spain in the population aged ≥15 years was estimated at 24.4% (daily and occasional use), with important variations according to sex, age, and AR. Overall, smoking prevalence was higher among men (25.6% versus 18.8% of women) and reached maximum values for those aged 25–54 years, close to 30% regardless of sex. While Galicia was the AR with the lowest prevalence (18.3%), Asturias registered the highest prevalence (27.7%). In all ARs, smoking was, in all cases, more prevalent among men, though in regions such as Navarre, the male to female prevalence ratio was estimated at 1.7 and in Castile and Leon at 1.1.

These data highlight the great heterogeneity in the epidemiology of smoking at a territorial level, thereby making it necessary to have age-related prevalence estimates for each sex at a subnational level. Such estimates would not only allow for a detailed characterization of the smoking epidemic but would also improve surveillance and help draw up and implement context-specific prevention policies. Owing to the NHS’s sample size, prevalence cannot be estimated for ARs by sex and age, separately. With regard to this aspect, a key factor in the analysis of the smoking epidemic, we only have information on some ARs that either have risk behavior surveillance systems or conduct their own health surveys. Two examples are the Catalan Health Interview Survey (*Encuesta de Salud de Catalu*ñ*a/ESCA*)^3^ and the Galician Risk Behavior Data System (*Sistema de Informaci*ó*n sobre Conductas de Riesgo en Galicia/SICRI*)^4^. The design of these surveys ensures representativeness in terms of sex, age, and both variables in the respective regions. For instance, the SICRI data have previously been used to estimate the prevalence of exposure to secondhand smoke in different scenarios by sex and age^5^.

Though theoretically ideal, having surveillance systems or health surveys at a subnational level is rare. Hence, in order to have detailed prevalence by sex and age, small-area estimation (SAE) methods could provide an efficient alternative^6-10^. Based on data collected in the NHS 2017, a small-area model was fitted for Spain, from which smoking prevalence at an AR level can be obtained by sex and age^1^. In comparison with the direct estimator, the small-area model yielded better precision in the estimates, with a mean error reduction of 26% for smokers and ex-smokers, and 25% for never smokers. The comparison between model-based estimates and direct estimates can be performed at different levels of aggregation on the same data source^1,11,12^ or against an external data source. Therefore, as a next step, this model should be externally validated by comparing its results with those obtained from the ESCA and SICRI for 2017. The aim of this study was thus to complete the validation of the previously fitted small-area model^1^.

## METHODS

To validate the previously fitted model^1^, smoking prevalence obtained with the SAE model at a subnational level, based on data collected in the NHS 2017, was compared against prevalence calculated with the direct estimator derived from the ESCA 2017 and SICRI 2017.

### Data sources

The smoking data were drawn from three surveys (NHS, ESCA and SICRI, 2017). All three included questions related to smoking, which were used to create the variable smoker status. For study purposes, a smoker (S) was defined as anyone who was smoking at the time of the survey, an ex-smoker (ExS) was defined as anyone who had smoked at some point in his/her lifetime but had already quit, and a never smoker (NS) was defined as anyone who had never smoked. Table 1 lists the verbatim questions included in each survey.

**Table 1:** Questions and responses about tobacco use included in the ENSE, ESCA and SICRI, in 2017, and used for the classification of interviewed individuals according to their smoking status

*Survey*	*Smoking status*
**ENSE 2017**	
**Q1. Could you tell me if you smoke?**	
Yes, I smoke daily	Smoker
Yes, I do smoke, but not daily	
I do not currently smoke, but I have smoked before	Ex-smoker
I do not smoke, and I have never smoked on a regular basis	Never smoker
**ESCA 2017**	
**Q1. Of the following situations, which best des cribes your smoking behavior?**	
You currently do not smoke at all	Go to Q2
You currently smoke occasionally (less than once a day)	Smoker
You currently smoke every day	
**Q2. In the past, did you smoke?**	
Never smoked at all	Never smoker
Had smoked less than once a day for 6 months or more	Ex-smoker
Had smoked less than once a day for less than 6 months	
Has smoked daily 6 months or more	
Had smoke daily for less than 6 months	
**SICRI 2017**	
**Q1. Have you ever smoked in your life?**	
Yes, daily	Go to Q2
Yes, occasionally	
No, never	Never smoker
**Q2. Do you currently smoke?**	
Daily	Smoker
Occasionally, at least once a week	
Sporadically, less than once a week	
Never	Ex-smoker

*Spanish National Health Survey 2017*

The small-area model was fitted based on data sourced from the NHS 2017^2^. This survey was conducted on a sample of 29195 people, 23089 of whom were aged ≥15 years and residing in main family dwellings nationwide. Data were collected by computer-assisted personal interviewing from October 2016 through October 2017. The sample was selected by three-stage stratified sampling, with the sampling units being first-, second- and third-stage census sections, main family dwellings, and one adult per household, respectively.

Based on the 17 ARs and the Autonomous Cities of Ceuta and Melilla considered jointly, sex and age (15–34, 35–54, 55–64, 65–74, and ≥75 years)^1^, 180 units of analysis or areas were defined. Then, the SAE model was applied to estimate the prevalence of S, ExS and NS in each area in 2017.

To validate the model, we used the results relating to Galicia and Catalonia, which correspond to 20 areas defined on the basis of the two ARs, sex, and five age groups considered.

In the case of Catalonia and Galicia, the NHS 2017 sample sizes for individuals aged ≥15 years were 2363 and 1335, respectively.

*Catalonian Health Survey 2017*

This survey^3^ was conducted on a sample of 3780 non-institutionalized residents in the AR of Catalonia, who were aged ≥15 years and responded to a direct questionnaire. Data were collected through computer-assisted personal interviewing from January through December 2017. The sample was selected by three-stage stratified sampling, with the sampling units being first-, second- and third-stage healthcare management areas, towns, and individuals (selected by stratified random sampling by 13 age groups and sex), respectively.

*Galician Risk Behavior Data System 2017*

This survey^4^ was conducted on a sample of 7841 persons aged >15 years residing in the AR of Galicia, and included in the Health Card Register (*Registro de Tarxeta Sanitaria*). Data were collected by computer-assisted telephone interviewing from January through December 2017. The sample was selected by stratified random sampling by sex and age.

### Statistical analysis

Based on microdata sourced from the ESCA and SICRI for 2017, which were available on the websites of the Government of Catalonia, Ministry of Health (*Departamento de Salud, Generalitat de Catalunya*)^3^ and Galician Regional Public Health Authority (*Dirección Xeral de Saúde Pública*)^4^, respectively, the prevalences of S, ExS and NS were calculated by sex and age (15–34, 35–54, 55–64, 65–74, and ≥75 years)^1^, by applying a weighted ratio estimator (direct estimator):


$$\hat{p}=\frac{\Sigma_{i}W_{i}X_{i}}{\Sigma_{i}W_{i}}$$ (1)

where *i* is the individual, *X_i_* is the value of the characteristic estimated (S, ExS or NS) in individual *i*, which takes values 0–1, and *W_i_* is the sampling weight of individual *i*. We calculated the variance of this estimator using a linear approach in Taylor’s series and, on the basis of this, then obtained the coefficients of variation (CVs).

*Mixed multinomial small-area model with random area effect*

Using the NHS 2017, we obtained the prevalence of S, ExS and NS for the 180 areas ([17 ARs + Ceuta and Melilla] × 2 sexes × 5 age groups), applying a mixed multinomial small-area model with a random area effect^13^. This model uses aggregated data on smoking sourced from surveys, such as the NHS, and auxiliary information sourced from administrative records. The response variable is a vector with the number of S, ExS and NS in each area. Smoking-related variables were chosen as auxiliary data. The model is expressed as:


$$P_{dk}=\frac{exp(\eta_{dk})}{1+exp(\eta_{d1})+exp(\eta_{d2})}$$

$$\eta_{dk}=\log\frac{p_{dk}}{p_{d3}}=x_{dk}\beta_{k}+u_{dk},d=1,...,D\text{ and }k=1,2$$ (2)

where *p_dk_* is the prevalence of each category *k* corresponding to area *d*, *x_dk_=(x_dk1_,…, x_dkrk_)*' is the set of covariates corresponding to category *k* and area *d*, and *β_k_=(β_k1_,…, β_krk_)*' is the vector of regression parameters. The subscript *k* refers to the category of S (*k*=1) or ExS (*k*=2). The third category of NS (*k*=3) is taken as reference, so that *p_d3_=1-p_d1_-p_d2_.* The model also considers the random effect *u_dk_* associated with area *d* and category *k*.

The CVs for the small-area model were calculated as the square root of the mean squared error. Further information about the model fitted can be found in Santiago et al.^1^.

To evaluate the quality of the estimates yielded by the small-area model, the estimates from the NHS for the areas of Catalonia and Galicia were compared against the direct estimates obtained from the ESCA and SICRI for 2017. For comparison purposes, two aspects were considered: 1) the similarity between the specific estimates obtained with both methods; and 2) the precision of the estimates. With respect to the first aspect, we calculated the intraclass correlation coefficient (ICC) and its 95% confidence interval (95% CI). The ICC measures the degree of concordance between two or more quantitative variables. Assuming a one-way random-effects model, the individual absolute-agreement ICC^14^ between two methods can be estimated as:


$$I\hat{C}C=\frac{BMS\text{-}WMS}{BMS+WMS}$$ (3)

$$WMS=\Sigma_{d}\Sigma_{m}\frac{{\left(p_{dm}\text{-}{\bar{p}}_{d.}\right)}^{2}}{D},BMS=\Sigma_{d}\frac{{\left({\bar{p}}_{d}\text{-}{\bar{p}}_{d..}\right)}^{2}}{D\text{-1}},d=1,...,D\text{ and }m=1,2$$

where *p_d._=*∑*_m_ p_dm_/2* and *p_.._=*∑*_d_ p_d._/D. WMS* is the mean squares within areas, *BMS* is the mean squares between areas, *d* is the area, and *m* is the method. The lower and upper limits on 95% CI for *IĈC* are:


$$\left(\frac{F_{L}\text{-1}}{F_{L}},\frac{F_{U}\text{-1}}{F_{U}}\right)$$

where $F_{L}=\frac{BMS}{WMS\;F_{l}}$, $F_{U}=\frac{BMS\;F_{u}}{WMS}$, and *F_l_* and *F_u_* denote the 97.5 percentiles of the *F_D-1,D_* and *F_D,D-1_* distributions, respectively.

The ICC takes values 0–1, where 1 indicates perfect concordance. Following the guidelines of Cicchetti^15^, criteria and rules of thumb, a concordance ≥0.60 was deemed acceptable.

For each area, we also calculated the differences between the SAE and direct estimates, along with their 95% CI obtained by Wald’s method. Under independence, the variance of the difference between estimates is equal to the sum of the variances. Since the standard error (SE) is equal to the square root of the variance, applying the Wald’s method we have that the 95% CI = difference ± 1.96 SE. No significant differences were deemed to exist between the two methods if the 95% CI of the differences included zero. To evaluate the precision of the estimates, the CV was calculated for each method. CVs <30% were considered acceptable, taking into account the criteria applied by the National Center for Health Statistics^16^. All statistical tests were two tailed.

Model fitting was carried out with the *mme* package of R^17^, and statistical analyses were performed using the Stata IC v17 computer software package^18^.

## RESULTS

**Figure 1:**
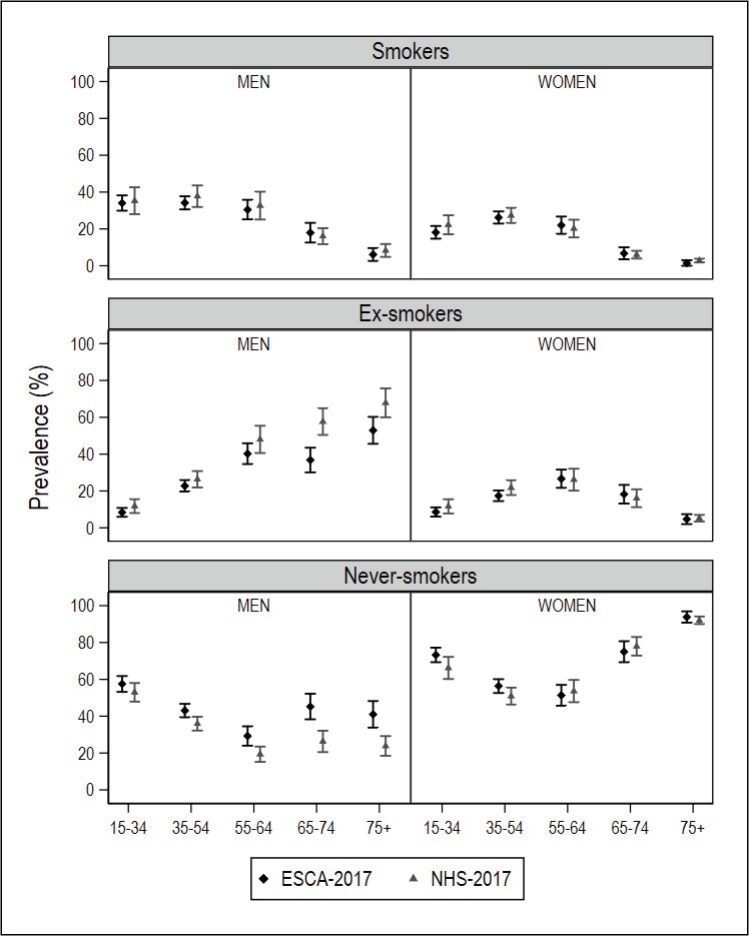
Prevalence of smokers, ex-smokers, and never smokers in 2017, by sex and age group, obtained using the National Health Survey with the small-area model, and using the ESCA (Catalonia) with the direct estimator, and their 95% confidence intervals

**Figure 2:**
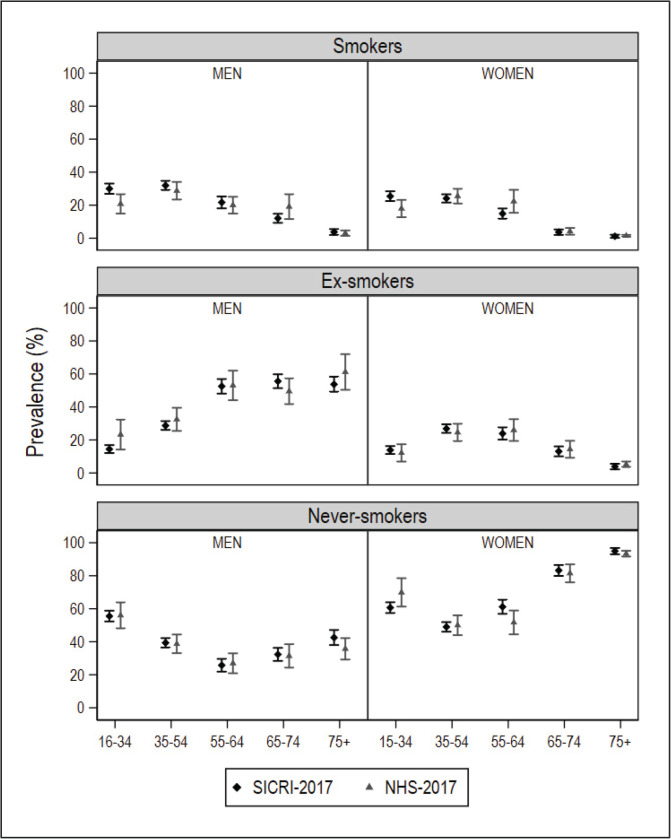
Prevalence of smokers, ex-smokers, and never smokers in 2017, by sex and age group, obtained using the National Health Survey with the small-area model, and using the SICRI (Galicia) with the direct estimator, and their 95% confidence intervals

**Figure 3:**
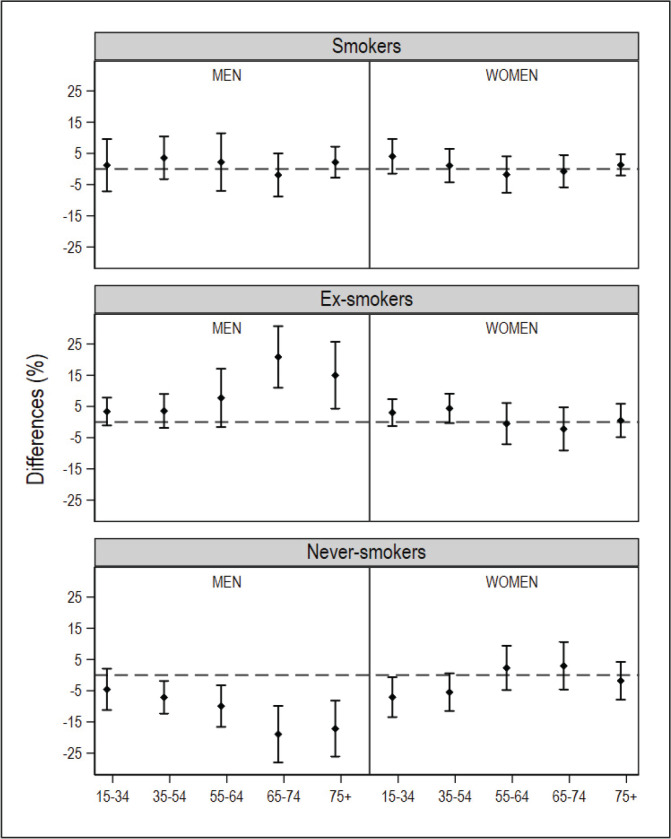
Differences in the prevalence of smokers, ex-smokers, and never smokers in 2017, by sex and age group, obtained using the National Health Survey with the small-area model, and using the SICRI (Galicia) with the direct estimator, and their 95% confidence intervals. The dotted horizontal line denotes zero

**Figure 4:**
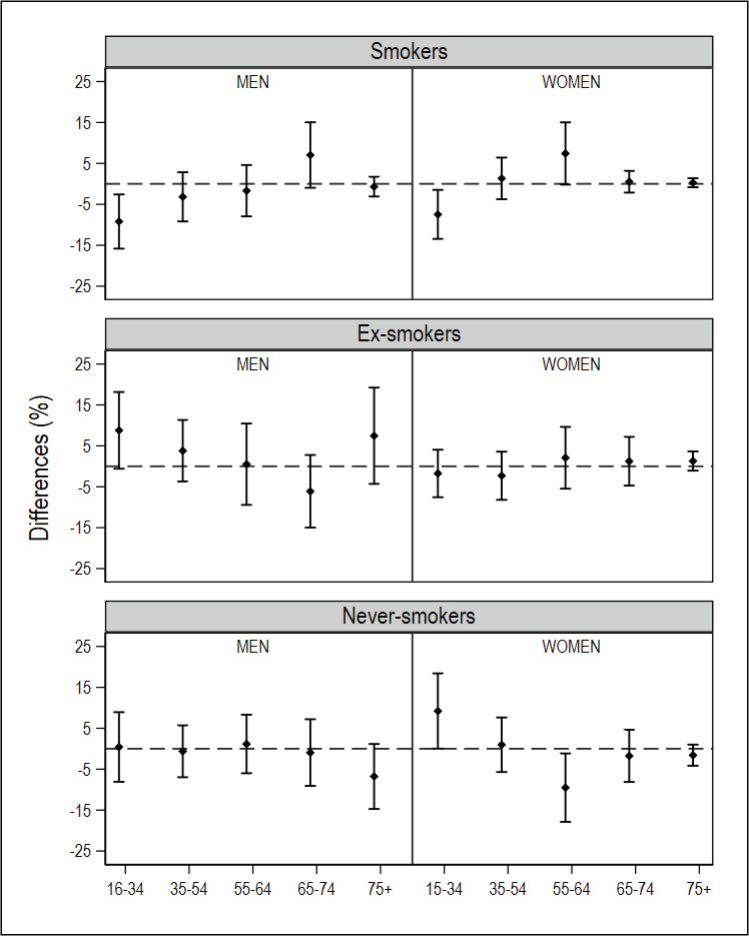
Differences in the prevalence of smokers, ex-smokers, and never smokers in 2017, by sex and age group, obtained using the National Health Survey with the small-area model, and using the ESCA (Catalonia) with the direct estimator, and their 95% confidence intervals. The dotted horizontal line denotes zero

After database cleaning, the small-area model was applied to the final NHS 2017 sample of 1334 individuals in Galicia and 2354 individuals in Catalonia. In the ESCA and the SICRI, there were no missing records, and the samples remained unaltered. The graphical comparisons of the prevalence estimated with the small-area model and the direct estimator for the ARs of Catalonia and Galicia, by sex and age, are shown in Figures 1 and 2. The estimates are shown in Supplementary file Tables S1 and S2. The differences between the SAE obtained with NHS data and with direct estimates sourced from the ESCA and SICRI, with their corresponding 95% CI, are shown in Figures 3 and 4.

The ICC between the SAE based on the NHS and the direct estimates for Catalonia based on the ESCA was 0.98 (0.94–0.99) for smokers, 0.88 (0.61–0.97) for ex-smokers, and 0.90 (0.68–0.97) for never smokers. In the case of Galicia, the ICC between the SAE and the direct estimates obtained from the SICRI was 0.88 (0.62–0.97) for smokers, 0.97 for ex-smokers (0.90–0.99), and 0.98 for never smokers (0.91–0.99). The estimated ICC was ≥0.88, exceeding the 0.60 taken as a reference.

In comparison with the ESCA-based prevalence for Catalonia, those yielded by the small-area model with NHS data displayed a similar precision. While the prevalence of smokers by sex and age drawn from both surveys was similar, in the case of men who were ex-smokers or never smokers, the differences were observed to increase with age. Among women who were ex-smokers or never smokers, the main differences were found in the youngest age groups (Figure 1). With respect to the 95% CI of the differences between estimates, of the 10 intervals calculated, 10 included zero in smokers, 8 included zero in ex-smokers, and 5 included zero in never smokers (Figure 3).

In comparison with the SICRI-based prevalence for Galicia, those yielded by the small-area model with NHS data displayed a lower precision, reflected in wider confidence intervals. Concerning the estimates, the most marked differences were seen among smokers of both sexes aged 16–34 years, men ex-smokers, and women never smokers aged 16–34 years and 55–64 years (Figure 2). Of the ten 95% CI of the differences calculated, 8 contained zero in smokers, 10 contained zero in ex-smokers, and 8 contained zero in never smokers (Figure 4).

As regards the precision of the estimates, Supplementary file Figure S1 shows the CVs for the estimated prevalence of smokers, ex-smokers, and never smokers, applying the small-area model to NHS data and the direct estimator to ESCA and SICRI 2017 data. In the case of SICRI, women smokers aged ≥75 years have been removed from the boxplot in order to avoid distortions. In SICRI-2017, 512 women aged ≥75 years were interviewed, but only six were smokers. This translates into a low prevalence and a large CV for the older group of women smokers in Galicia. Thus, if their CV is taken into account, a distortion occurs in the boxplot, giving the wrong idea of the SICRI’s precision. In ex-smokers and never smokers, the CVs for the estimated prevalence were slightly higher with the small-area model. A greater similarity was seen between the CVs obtained from the ESCA and the NHS.

SAE based on the small-area model and direct estimates proved to be consistent in terms of the median and interquartile range (IQR) for the prevalence of smokers and ex-smokers in Galicia, with greater differences being in evidence between the IQRs of ex-smokers and never smokers in Catalonia. The range of prevalence of smokers and never smokers in Galicia was narrower with the small-area model than with the direct estimator (Supplementary file Table S3).

## DISCUSSION

The results of this study highlight the fact that small-area models can furnish reliable estimates at a subnational level in cases where national survey data are not representative by sex and age, thus making such models a useful alternative for acquiring context-specific information on health policies. In this study, the estimated prevalence of smokers, ex-smokers and never smokers by sex and age, obtained with the small-area model and with the direct estimator for Galicia, were generally similar. The main differences are found in women smokers and never smokers aged 16–34 years and 55–64 years, and in men smokers aged 16–34 years and 65–74 years and ex-smokers aged 16–34 years. In the case of Catalonia, the small-area method offers similar estimates to those obtained with the direct estimator for women as well as men smokers. Among men ex-smokers aged ≥55 years, the small-area method tends to overestimate prevalence compared with the direct estimator, whereas among never smokers the opposite effect is seen. In terms of precision of estimates, in general, the degree of precision afforded by the small-area method is lower than that of direct estimator when applied to data on Galicia, but is very similar in the case of Catalonia. In addition, the differences between the precision of the two methods are greater in smokers, followed by ex-smokers, and smaller in never smokers. These aspects could be due to the sample size and the magnitude of the prevalence.

The sample size of the SICRI was 5.9 times that of the NHS for Galicia in 2017. The sample size difference between both surveys, by sex and age, ranges from a minimum of 361 individuals in the group of women aged 65–74 years and ≥75 years to a maximum of 1213 individuals in the group of women aged 16–34 years. In the case of the ESCA, the sample size is 1.7 times that of the NHS for Catalonia. The sample size difference ranges from a minimum of 16 individuals in the group of women aged 65–74 years to a maximum of 317 individuals in the group of men aged 15–34 years. Our results indicate that the small-area model, with almost half the sample, can offer a degree of precision in estimates similar to that obtained with the direct estimator applied to a representative sample at an area level.

Analysis of the CVs shows a single value >30%, corresponding to the group of smokers, when using SICRI-based data: this is associated with women smokers aged ≥75 years in Galicia, with a CV of 39.6%. In 2017, the SICRI compiled data on only six women smokers aged ≥75 years, due to the anecdotal nature, until now, of smoking prevalence among women in this age group. These data reflect the fact that direct estimates, though asymptotically unbiased, tend to be somewhat unreliable in cases where the sample size is small. In this same group, the CV associated with the small-area model is 20.6%, and the sample size is zero. Small-area methods enable reliable estimates to be obtained, even in cases where the sample size is so small that direct estimates would display great variability or not even be calculable. In such cases, SAEs can be obtained by using the synthetic part of the linear predictor of the small-area model, as was done in Santiago et al.^1^.

Many studies have applied small-area models to estimate the prevalence of health indicators at a subnational level, though the methods vary from one study to another^8,19-21^. Some studies include external validation of the method used^22-24^. For this purpose, a comparator is needed, which is deemed to be the best direct estimation available at an area level and is based on a sufficiently large sample size to ensure representativeness. This can be achieved by defining areas with sufficient size for a single year, as in the case of the ESCA and SICRI, through pooling several survey years or increasing the sample size.

For external validation, two aspects must be considered: the similarity between the estimates obtained with the methods to be compared, and the precision of the estimates. To evaluate the first aspect, the estimates of the direct estimator and small-area model are usually related by means of a coefficient. In some studies, the Pearson correlation coefficient is used to relate estimates obtained with the methods sought to be compared. In our study, however, the ICC was used to measure the degree of concordance between the direct estimates and those obtained with the small-area model. This coefficient gives a global quantification of the extent to which the estimates obtained with both methods agree. The Pearson correlation coefficient, for its part, provides a measure of the direction and strength of linear association between two quantitative variables but does not indicate the degree of concordance between them. Hence, if one of the variables systematically deviates from the other by the same margin, the correlation between the two will be perfect, but the variables will not be concordant. Our results show a concordance between the prevalence of smokers, ex-smokers, and never smokers ≥0.88.

Furthermore, it is common practice to analyze whether SAEs are contained in the 95% CI of the prevalence estimated with the direct estimator as part of the external validation process. Due to being obtained with different methods, the estimates are subject to different errors, and each has its own CI. In this respect, we calculated the difference between the estimates obtained with both methods and ascertain their respective CI. Hence, analyzing whether the CI includes zero makes it possible to determine, for any given area, whether there are significant differences between the SAEs and the prevalence obtained with the direct method. In our case, 81.6% of the intervals analyzed include zero, meaning that there are no significant differences between the estimates obtained with both methods in more than 8 out of 10 areas analyzed. Furthermore, in two of the areas, the lower limits of the 95% CI of the differences lie very close to zero, with these being women who were never smokers in Catalonia and Galicia aged 15–34 years, with differences of 9.22 (0.02–18.42) and -7.08 (-13.48 – -0.67), respectively.

### Limitations

This study has limitations related to the data sources. Some auxiliary variables used in the small-area model were drawn from the 2011 Census because more up-to-date quality data were unavailable. Due to the time difference between 2011 and 2017, it is possible that some of the covariates may not adequately reflect their distribution in the year of estimation, which could affect the results of the small-area model. This study’s main advantage lies in validating the small-area model in two ARs that display mutual social and economic differences and different trends in the smoking epidemic. For instance, the SICRI and ESCA data show how the incorporation of women into smoking took place in the two territories at different points in time. In Galicia, a pronounced downward trend was observed in the prevalence of middle-aged women who were never smokers (45–64 years) across the period 2005–2017. This same trend is likewise observed in Catalonia but at more advanced ages (65–74 years). In the latter AR, the percentage of women who were never smokers dropped by 20 points, in the period from 1994 through 2017.

## CONCLUSIONS

External validation is fundamental for evaluating the small-area model and its application to other contexts. Our results suggest that, despite there being certain differences between the estimates obtained with the two methods in some of the areas analyzed, SAEs nonetheless display good concordance with direct estimates and are reliable. They can thus be used to characterize geographical variations at a subnational level as well as to quantify differences in the prevalence of risk factors in cases where there are no survey data that guarantee representativeness. The small-area model has been seen to be capable of obtaining estimates with similar precision as the direct estimator, even in cases where the sample size is reduced to almost half, as in the case of Catalonia. These results indicate that the small-area model applied to national survey data yields valid estimates of smoking prevalence by sex and age at the AR level. Accordingly, these models could be applied to a single year’s data from a national health survey of any country to characterize the status of different risk factors in a population at a subnational level and so help configure context-specific health interventions.

## Data Availability

The data supporting this research are available from the following sources: https://www.sergas.es/Saude-publica/SICRI-2017, https://scientiasalut.gencat.cat/handle/11351/4125, https://www.sanidad.gob.es/estadEstudios/estadisticas/encuestaNacional/home.htm
