# Supplementary material for: Validation of a small-area model for estimation of smoking prevalence at a subnational level
Source: Tob Induc Dis. 2023 Sep 1;21:112. doi: 10.18332/tid/169683 (PMC10472341; doi:10.18332/tid/169683)
Supplement: Supplementary file 1 [file TID-21-112-s1.pdf]

Validation of a small-area model for estimation of smoking prevalence at a subnational level

Supplementary Material

Table S1. Prevalence of smokers, ex-smokers, and never-smokers obtained with the small-area model (National Health Survey 2017) and with the direct estimator (ESCA 2017) for the Autonomous Region of Catalonia, by sex and age group, and their 95% confidence intervals (95%CI)

**ESCA 2017**	**Smokers**		**Ex-smokers**		**Never-smokers**	
**Sex and age**	**%**	**95%CI**	**%**	**95%CI**	**%**	**95%CI**
Men 15-34 years	34.1	29.9 - 38.2	8.4	6.0 – 10.8	57.5	53.2 – 61.8
Men 35-54 years	34.2	30.6 – 37.7	22.8	19.7 – 25.9	43.1	39.4 – 46.7
Men 55-64 years	30.5	25.2 – 35.8	40.2	34.6 – 45.9	29.3	24.0 – 34.5
Men 65-74 years	18.0	12.6 – 23.3	36.8	30.1 – 43.5	45.2	38.3 – 52.5
Men 75 years and over	6.1	2.6 – 9.6	52.9	45.6 – 60.2	41.0	33.8 – 48.2
Men >=15 years	29.7	27.6 – 31.8	25.2	23.2 – 27.1	45.1	42.9 – 47.4
Women 15-34 years	18.2	14.7 – 21.6	8.6	6.1 – 11.1	73.3	69.3 – 77.2
Women 35-54 years	26.2	22.9 – 29.5	17.4	14.5 – 20.3	56.4	52.6 – 60.1
Women 55-64 years	22.0	17.3 – 26.7	26.7	21.7 – 31.6	51.3	45.7 – 57.0
Women 65-74 years	6.7	3.5 – 10.0	18.3	13.2 – 23.3	75.0	69.3 – 80.7
Women 75 years and over	1.4	0.0 – 3.0	4.7	2.0 – 7.4	93.8	90.8 – 96.9
Women >=15 years	18.5	16.7 – 20.2	15.2	13.5 – 16.8	66.4	64.3 – 68.5
All 15-34 years	26.2	23.4 – 28.9	8.5	6.8 – 10.2	65.3	62.3 – 68.3
All 35-54 years	30.3	27.9 – 32.7	20.2	18.0 – 22.3	49.5	46.9 – 52.2
All 55-64 years	26.1	22.6 – 29.6	33.3	29.5 – 37.0	40.6	36.7 – 44.6
All 65-74 years	11.9	8.8 – 15.0	26.8	22.6 – 31.1	61.3	56.6 – 65.9
All 75 years and over	3.4	1.6 – 5.1	24.4	20.3 – 28.6	72.2	67.9 – 76.5
All	24.0	22.6 – 25.3	20.1	18.8 – 21.4	55.9	54.3 – 57.5
**Small-area model**	**Smokers**		**Ex-smokers**		**Never-smokers**	
**Sex and age**	**%**	**95%CI**	**%**	**95%CI**	**%**	**95%CI**
Men 15-34 years	35.3	28.0 - 42.6	11.8	8.0 - 15.5	53.0	47.9 - 58.0
Men 35-54 years	37.7	31.9 - 43.6	26.3	21.9 - 30.8	35.9	32.2 - 39.7
Men 55-64 years	32.7	25.1 - 40.2	48.0	40.6 - 55.4	19.4	15.2 - 23.5
Men 65-74 years	16.0	11.7 - 20.4	57.6	50.4 - 64.9	26.3	20.5 - 32.1
Men 75 years and over	8.3	4.8 - 11.8	67.9	60.0 - 75.7	23.8	18.5 - 29.2
Men >=15 years	31.5	28.2 - 34.8	32.4	29.9 - 34.9	36.1	33.8 - 38.3
Women 15-34 years	22.2	17.1 - 27.4	11.6	7.8 - 15.5	66.2	60.2 - 72.2
Women 35-54 years	27.3	23.2 - 31.4	21.8	17.8 - 25.8	50.9	46.3 - 55.5
Women 55-64 years	20.2	15.4 - 25.0	26.1	20.2 - 32.1	53.6	47.6 - 59.7
Women 65-74 years	6.0	3.9 - 8.1	16.0	11.2 - 20.9	78.0	72.9 - 83.0
Women 75 years and over	2.8	1.8 - 3.8	5.2	3.4 - 7.0	92.0	90.0 - 94.1
Women >=15 years	19.4	17.3 - 21.5	17.1	15.1 - 19.1	63.5	61.0 - 66.0
All 15-34 years	28.8	24.3 - 33.3	11.7	9.0 - 14.4	59.5	55.6 - 63.4
All 35-54 years	32.6	29.0 - 36.2	24.1	21.1 - 27.1	43.3	40.3 - 46.2
All 55-64 years	26.2	21.8 - 30.7	36.7	32.0 - 41.4	37.0	33.3 - 40.7
All 65-74 years	10.7	8.3 - 13.0	35.4	31.1 - 39.6	53.9	50.1 - 57.8
All 75 years and over	4.9	3.4 - 6.4	29.6	26.4 - 32.9	65.5	63.0 - 67.9
All	25.3	23.4 - 27.2	24.6	23.0 - 26.2	50.1	48.5 - 51.8

Table S2. Prevalence of smokers, ex-smokers, and never-smokers obtained with the small-area model (National Health Survey 2017) and with the direct estimator (SICRI 2017) for the Autonomous Region of Galicia, by sex and age group, and their 95% confidence intervals (95%CI)

**SICRI 2017**	**Smokers**		**Ex-smokers**		**Never-smokers**	
**Sex and age**	**%**	**95%CI**	**%**	**95%CI**	**%**	**95%CI**
Men 16-34 years	30.0	26.9 - 33.1	14.5	12.1 - 16.9	55.5	52.3 - 58.8
Men 35-54 years	31.9	29.2 - 34.7	28.7	26.1 - 31.4	39.3	36.5 - 42.2
Men 55-64 years	21.7	18.1 - 25.3	52.5	48.1 - 56.9	25.8	21.9 - 29.6
Men 65-74 years	12.1	9.3 - 14.8	55.6	51.4 - 59.8	32.3	28.4 - 36.3
Men 75 years and over	3.7	2.0 - 5.5	53.7	49.2 - 58.3	42.5	38.0 - 47.1
Men >=16 years	23.8	22.3 - 25.2	36.6	35.1 - 38.2	39.6	38.0 - 41.2
Women 16-34 years	25.4	22.5 - 28.4	13.9	11.5 - 16.3	60.7	57.4 - 63.9
Women 35-54 years	24.1	21.6 - 26.6	26.9	24.3 - 29.5	49.0	46.1 - 51.9
Women 55-64 years	14.9	11.8 - 18.0	23.9	20.2 - 27.6	61.2	56.9 - 65.4
Women 65-74 years	3.7	2.0 - 5.3	13.1	10.1 - 16.1	83.2	79.9 - 86.5
Women 75 years and over	1.2	0.2 - 2.1	3.9	2.2 - 5.6	94.9	93.0 - 96.8
Women >=16 years	16.0	14.8 - 17.2	18.3	17.0 - 19.6	65.7	64.2 - 67.2
All 16-34 years	27.7	25.6 - 29.9	14.2	12.5 - 15.9	58.1	55.8 - 60.4
All 35-54 years	28.0	26.2 - 29.9	27.8	25.9 - 29.7	44.2	42.1 - 46.2
All 55-64 years	18.2	15.8 - 20.6	37.7	34.8 - 40.6	44.1	41.2 - 47.1
All 65-74 years	7.5	5.9 - 9.0	32.4	29.8 - 35.1	60.1	57.3 - 62.8
All 75 years and over	2.2	1.3 - 3.1	23.6	21.3 - 26.0	74.2	71.8 - 76.6
All	19.7	18.8 - 20.6	27.0	26.0 - 28.0	53.3	52.2 - 54.4
**Small-area model**	**Smokers**		**Ex-smokers**		**Never-smokers**	
**Sex and age**	**%**	**95%CI**	**%**	**95%CI**	**%**	**95%CI**
Men 16-34 years	20.8	14.9 - 26.6	23.2	14.2 - 32.3	56.0	48.1 - 63.8
Men 35-54 years	28.8	23.4 - 34.1	32.5	25.5 - 39.5	38.7	33.1 - 44.4
Men 55-64 years	20.0	14.9 - 25.1	53.0	44.1 - 62.0	26.9	20.9 - 33.0
Men 65-74 years	19.1	11.6 - 26.6	49.5	41.7 - 57.3	31.4	24.3 - 38.5
Men 75 years and over	3.1	1.4 - 4.7	61.2	50.4 - 72.0	35.7	29.2 - 42.2
Men >=16 years	21.3	18.6 - 24.0	39.2	35.3 - 43.2	39.5	36.3 - 42.6
Women 16-34 years	18.0	12.7 - 23.2	12.1	6.9 - 17.4	69.9	61.3 - 78.5
Women 35-54 years	25.4	21.0 - 29.9	24.6	19.3 - 29.8	50.0	44.0 - 56.0
Women 55-64 years	22.3	15.4 - 29.3	26.0	19.4 - 32.6	51.7	44.5 - 58.9
Women 65-74 years	4.2	2.1 - 6.2	14.3	9.2 - 19.5	81.5	76.0 - 86.9
Women 75 years and over	1.4	0.9 - 2.0	5.2	3.6 - 6.9	93.4	91.6 - 95.1
Women >=16 years	16.5	14.3 - 18.6	17.6	15.1 - 20.0	66.0	63.0 - 69.0
All 16-34 years	19.4	15.5 - 23.3	17.8	12.5 - 23.1	62.8	57.0 - 68.6
All 35-54 years	27.1	23.6 - 30.6	28.5	24.1 - 32.9	44.4	40.3 - 48.5
All 55-64 years	21.2	16.9 - 25.6	39.1	33.6 - 44.6	39.7	34.9 - 44.4
All 65-74 years	11.2	7.5 - 14.8	30.8	26.2 - 35.3	58.1	53.7 - 62.5
All 75 years and over	2.1	1.3 - 2.8	26.8	22.5 - 31.1	71.1	68.4 - 73.8
All	18.8	17.1 - 20.5	27.9	25.6 - 30.2	53.3	51.2 - 55.5

Table S3. Summary statistics for prevalence of smokers, ex-smokers, and never-smokers estimated using the small-area model with data sourced from the National Health Survey 2017, and using the direct estimator with data sourced from the SICRI and ESCA 2017

	Q1	Median	Q3	IQR	Minimum	Maximum	Mean
**Smokers**							
Small-area model Galicia	4.19	19.57	22.32	18.13	1.44	28.76	16.31
SICRI 2017	3.70	18.30	25.40	21.70	1.20	31.90	16.87
Small-area model Catalonia	8.28	21.21	32.67	24.39	2.76	37.74	20.85
ESCA 2017	6.70	20.10	30.50	23.80	1.40	34.20	19.74
**Ex-smokers**							
Small-area model Galicia	14.33	25.29	49.51	35.18	5.20	61.21	30.18
SICRI 2017	13.90	25.40	52.50	38.60	3.90	55.60	28.67
Small-area model Catalonia	11.77	23.97	47.97	36.20	5.23	67.89	29.25
ESCA 2017	8.60	17.85	36.80	28.20	4.70	52.90	22.68
**Never-smokers**							
Small-area model Galicia	35.73	50.83	69.88	34.15	26.92	93.35	53.51
SICRI 2017	39.30	52.25	61.20	21.90	25.80	94.90	54.44
Small-area model Catalonia	26.30	51.93	66.17	39.87	19.35	92.01	49.90
ESCA 2017	43.10	53.85	73.30	30.20	29.30	93.80	56.59

Fig. S1 Distribution of the coefficients of variation of the direct estimators and the small-area model for prevalence of smokers, ex-smokers, and never-smokers in 2017

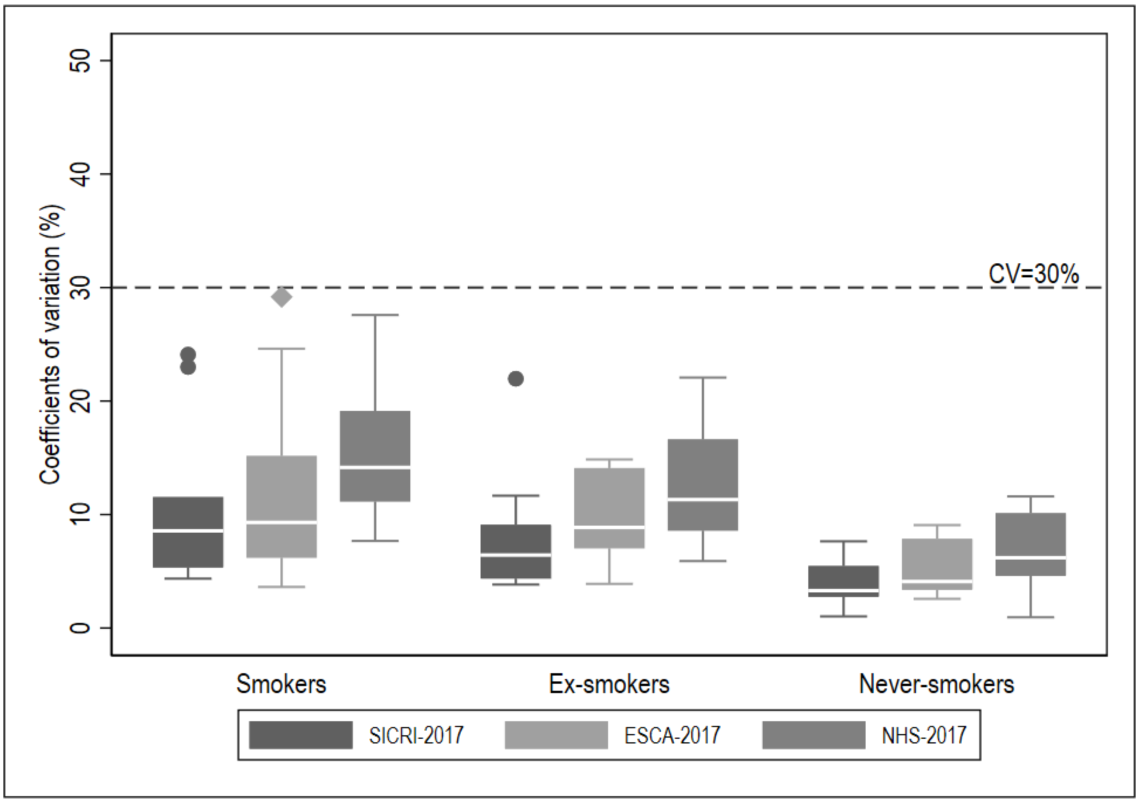

Note: Women smokers aged 75 years and over have been removed from SICRI-2017 to avoid distortions

©2023 Guerra-Tort C. et al.
